# Authority cues and metacognitive miscalibration in linguistic judgment: evidence from an English–Chinese revision paradigm

**DOI:** 10.3389/fpsyg.2026.1877483

**Published:** 2026-07-23

**Authors:** Chen Chen, Ning Ren, Zikai Guo

**Affiliations:** School of Foreign Studies, Xi'an Medical University, Xi'an, China

**Keywords:** authority cues, confidence calibration, linguistic judgment, metacognition, source credibility, translation revision

## Abstract

**Introduction:**

Linguistic judgment rarely occurs in a social vacuum. This study examined whether social authority cues reshape the alignment between confidence and decision accuracy, known as metacognitive calibration, and subsequently influence linguistic revision behavior.

**Methods:**

Using an English-Chinese revision paradigm, participants evaluated translation segments labeled as produced by an “expert,” a “peer,” or an anonymous source. We examined perceived source credibility, linguistic quality judgment, metacognitive calibration, and revision behavior, including under-editing of erroneous segments and over-editing of acceptable segments.

**Results:**

Authority cues significantly influenced perceived credibility and linguistic quality judgments. Expert labels increased overconfidence when participants evaluated erroneous segments and were associated with a higher likelihood of under-editing. Conversely, peer labels induced skepticism and were associated with unnecessary revisions to acceptable segments. Mediation analysis indicated that calibration bias partly accounted for the associations between authority labels and revision behavior. However, because confidence and revision decisions were measured within the same segment-level task, these indirect effects should be interpreted as statistically consistent explanatory pathways rather than definitive evidence of temporally ordered causal mechanisms.

**Discussion:**

Social authority cues may affect not only linguistic judgments but also individuals' monitoring of the reliability of those judgments. These findings demonstrate the vulnerability of metacognitive calibration to source information and provide a psychological framework for understanding biased intervention in professional communication.

## 1 Introduction

Linguistic judgment is not made in a social vacuum. When individuals evaluate whether a sentence, expression, or translation segment is acceptable, they may respond not only to the linguistic material itself but also to contextual cues surrounding it. Among these cues, perceived source identity is especially important. A text attributed to an expert may be evaluated differently from the same

text attributed to a peer or an anonymous source. This possibility is consistent with classic source credibility research, which shows that perceived expertise and credibility can shape message evaluation (Hovland and Weiss, 1951; Pornpitakpan, 2004), and with heuristic-processing accounts suggesting that source cues may guide judgment when decision makers face uncertainty (Chaiken, 1980; Meinert and Krämer, 2022). From this perspective, authority cues are not external decorations attached to language; they may become social inputs to linguistic judgment.

A second psychological issue concerns metacognitive confidence. In language-based decisions, individuals must not only decide whether a linguistic item is correct, but also estimate how reliable their own judgment is. Metacognitive theories treat confidence as a judgment about the reliability of one's own decision (Koriat, 2012; Fleming and Lau, 2014). Ideally, confidence should be aligned with accuracy: people should be more confident when their judgments are correct and less confident when they are wrong. However, confidence can be distorted by non-diagnostic cues, leading to overconfidence, underconfidence, or broader confidence–accuracy miscalibration (Maniscalco and Lau, 2012; Shekhar and Rahnev, 2021; Fleming, 2024; Rapp and Withall, 2024). This raises a key question for the psychology of language: can social authority cues disrupt metacognitive calibration during linguistic judgment?

Translation revision offers a useful paradigm for addressing this question. In a revision task, participants compare a source text with a target-language rendering, decide whether a segment should be changed, and implement or withhold a revision. This task therefore combines language comprehension, judgment under uncertainty, confidence monitoring, and behavioral control. Prior research has treated translation revision as a decision-making process rather than a purely mechanical correction activity (Shih, 2015; Cao et al., 2023). Recent work on post-editing has also shown that source beliefs can bias editing behavior; for example, perceived human vs. AI source labels can shape trust, quality judgment, and revision intensity (Cao and Zhou, 2026). However, less is known about whether human–human authority cues, such as expert vs. peer labels, distort the metacognitive calibration of linguistic judgment.

The present study addresses this gap by using an English–Chinese revision paradigm. Participants reviewed the same set of translation materials under three authority-cue conditions: expert translator, peer translator, and anonymous source. The materials contained both objectively erroneous segments and objectively acceptable segments, allowing revision behavior to be evaluated against an objective standard. For each segment, participants made a revision decision and reported their confidence. This design allowed us to examine whether authority cues affect perceived source credibility, linguistic quality judgment, confidence–accuracy calibration, and revision behavior.

These hypotheses are intended to form a coherent explanatory sequence rather than a set of independent predictions. H1 and H2 examine whether experimentally assigned source labels alter perceived credibility and linguistic quality judgment. H3 examines whether those labels are associated with confidence–accuracy miscalibration during segment-level decisions. H4a and H4b examine the behavioral consequences of this miscalibration by distinguishing under-editing of erroneous segments from over-editing of acceptable segments. H5 then evaluates whether calibration bias statistically accounts for part of the association between authority cues and revision behavior.

The study makes three contributions. First, it extends source-credibility and authority-cue research to linguistic judgment by showing how perceived source identity can influence the evaluation of the same linguistic materials. Second, it extends metacognitive-confidence research by examining confidence–accuracy calibration in a linguistically complex judgment task rather than in a purely perceptual, memory-based, or general decision-making task. Third, it extends translation-revision research by treating revision behavior as a behavioral expression of metacognitive regulation, thereby distinguishing necessary correction from under-editing and unnecessary intervention from over-editing. By treating English–Chinese revision as a controlled linguistic judgment paradigm, this study provides a psychological account of how social authority cues are linked to confidence calibration and behavioral control in language-based decisions.

## 2 Literature review and hypothesis development

### 2.1 Linguistic judgment as a socially situated decision task

Linguistic judgment is often treated as a response to the internal properties of a linguistic stimulus, such as semantic adequacy, grammatical acceptability, pragmatic appropriateness, or stylistic fluency. However, judgment about language rarely occurs in a socially neutral environment. In many communicative contexts, individuals evaluate a text together with cues about its author, source, institutional status, or presumed expertise. These cues can shape not only whether a message is accepted, but also how much effort is invested in evaluating it and how confident individuals feel about their own judgments. Classic research on source credibility has shown that the same message may be evaluated differently when attributed to sources varying in credibility or expertise (Hovland and Weiss, 1951). Dual-process models of persuasion similarly suggest that source-related cues can function as heuristic or peripheral cues when individuals do not rely exclusively on message content (Chaiken, 1980; Petty and Cacioppo, 1986).

The present study extends this psychological logic to linguistic judgment. Specifically, it conceptualizes English–Chinese translation revision not merely as a translation-training activity, but as a controlled language judgment paradigm. In a revision task, participants must compare a source text with a target text, decide whether the target rendering is acceptable, determine whether a change is necessary, and regulate their confidence in that decision. Translation revision therefore combines language comprehension, error detection, evaluative judgment, and behavioral control. Prior work in translation revision has emphasized that revision involves more than mechanical correction; it requires comparing alternative renderings, identifying potential problems, and deciding whether a modification improves the target text (Robert, 2008; Mossop, 2014). Process-oriented studies have also described translation revision as a form of problem solving and decision making in which revisers must judge the appropriateness of competing solutions under uncertainty (Shih, 2015). More recent empirical work using eye tracking and screen recording has further demonstrated that translation revision can be studied through observable behavioral indicators, including attention allocation, revision time, revision location, and product quality (Cao et al., 2023).

This framing is important for the present research because the theoretical object is not translation quality per se. Rather, translation revision provides a task environment in which the accuracy of linguistic judgment can be objectively coded. Some segments contain genuine errors and require correction, whereas other segments are acceptable and should be left unchanged. This makes it possible to distinguish accurate revision from inaccurate revision, over-editing from necessary editing, and under-editing from appropriate restraint. By asking participants to report confidence in their own revision decisions, the task allows us to examine whether social authority cues distort metacognitive calibration in linguistic judgment. Thus, English–Chinese revision is used here as a behavioral paradigm for studying a psychological problem: how authority cues influence the relationship between confidence, accuracy, and action in language-based judgment.

### 2.2 Authority cues, source credibility, and source-based judgment

Authority cues refer to contextual signals that imply expertise, competence, professional status, or institutional legitimacy. In the present study, such cues are operationalized through translator identity labels. The same translated text may be described as produced by a senior professional translator, a student translator, or an anonymous translator. Because the objective linguistic material is held constant across cue conditions, any difference in judgment can be attributed to the perceived status of the source rather than to the actual quality of the translation.

It is important to clarify that the present study does not define the use of source labels as inherently irrational authority bias. In real-world language evaluation, source identity may provide a plausible cue about expected error probability. A translation attributed to an experienced professional may reasonably be expected to contain fewer errors, whereas a translation attributed to a student translator may reasonably be expected to contain more errors. Thus, participants' responses to expert and peer labels can be understood as heuristic source-based judgment or as prior adjustment under uncertainty. The critical question in the present experiment is whether such source-based expectations change judgment, confidence calibration, and revision behavior when the objective translation materials are held constant across label conditions.

The theoretical foundation for this manipulation comes from source credibility and heuristic processing research. Source credibility theory suggests that perceived expertise and trustworthiness can influence the acceptance and evaluation of information (Hovland and Weiss, 1951; Pornpitakpan, 2004). In the heuristic-systematic model, credibility cues may guide judgment when individuals rely on simple decision rules, such as “experts are likely to be correct” (Chaiken, 1980). Similarly, the elaboration likelihood model proposes that source expertise can operate as a peripheral cue under lower elaboration, or as a biasing factor that affects how content is processed under higher elaboration (Petty and Cacioppo, 1986). Recent work on the expertise heuristic also shows that expertise cues can accelerate credibility judgments and reduce decision effort, suggesting that individuals may use expertise as a shortcut when evaluating uncertain information (Meinert and Krämer, 2022).

These perspectives are directly relevant to linguistic judgment. When participants revise a translation, they may not always be fully certain whether a segment is genuinely erroneous or merely stylistically different. Under such uncertainty, a professional translator label may increase the prior expectation that the original translation is correct, whereas a student translator label may increase the prior expectation that the original translation is problematic. In the present counterbalanced design, however, these labels were experimentally assigned and were independent of actual segment quality. Therefore, when different labels lead to different credibility judgments, quality judgments, confidence ratings, or revision decisions for the same underlying materials, the effect is interpreted as a source-label-induced shift in judgment. In this sense, the term “bias” in the present manuscript refers to a systematic shift relative to identical materials, not to a claim that participants' use of source information was necessarily irrational.

Recent research in post-editing provides a closely related precedent. Scansani et al. (2019) experimentally examined whether translator trainees behaved differently when they believed they were revising human translation or post-editing machine translation. More directly, Cao and Zhou (2026) found that source beliefs about human vs. AI translation affected post-editing judgments and behavior: perceived AI labels were associated with lower trust, greater revision intensity, stronger error sensitivity, and more conservative quality ratings, even when translation quality was held constant. The present study follows the same general logic of source attribution but differs in two important ways. First, it does not examine human–machine attribution; it examines human–human authority attribution, namely expert vs. peer vs. anonymous source cues. Second, it does not treat labeling effects only as general judgment shifts; it asks whether source-based expectations are associated with changes in the confidence–accuracy relationship during linguistic judgment.

Accordingly, the first set of hypotheses concerns perceived authority and source-based judgment:

**H1**. Authority cues will affect perceived source credibility in linguistic judgment. Translations labeled as produced by a senior professional translator will be perceived as more authoritative and credible than the same translations labeled as produced by a peer/student translator or presented without identity information.

**H2**. Authority cues will influence linguistic quality judgments independently of objective translation quality. Expert-labeled translations will receive more favorable quality judgments, whereas peer/student-labeled translations will receive more skeptical quality judgments, relative to anonymous translations.

### 2.3 Metacognitive confidence and miscalibration in linguistic judgment

Although judgment bias is important, the central psychological mechanism in this study is metacognitive miscalibration. Metacognition refers to the monitoring and control of one's own cognitive processes (Nelson and Narens, 1990). In judgment tasks, one of its most important manifestations is confidence: the degree to which individuals believe that their own decision is correct. Confidence is not simply an affective reaction; it is a metacognitive estimate of decision reliability. A well-calibrated judge should be more confident when their judgment is correct and less confident when their judgment is incorrect. Miscalibration occurs when confidence and accuracy diverge, such as when individuals are highly confident in an erroneous decision or insufficiently confident in a correct decision.

Contemporary metacognition research distinguishes between absolute and relative aspects of metacognitive judgment. Absolute accuracy, often discussed in terms of calibration, refers to the extent to which confidence corresponds to actual correctness. A participant is well calibrated when high confidence is attached to correct judgments and low confidence is attached to incorrect judgments. In contrast, relative accuracy, often referred to as metacognitive resolution or sensitivity, concerns whether confidence ratings successfully discriminate correct from incorrect decisions across trials (Maniscalco and Lau, 2012; Fleming and Lau, 2014). Both aspects are relevant to linguistic judgment. In a language-revision task, absolute calibration is important because a reviser may be overly confident in leaving an erroneous segment unchanged or in revising an already acceptable segment. Relative accuracy is also important because a well-regulated reviser should assign higher confidence to correct language judgments than to incorrect ones.

The present study focuses primarily on absolute and directional calibration because the theoretical question concerns whether authority cues inflate or deflate confidence relative to the objective correctness of specific segment-level revision decisions. The key issue is not only whether participants can distinguish correct from incorrect judgments in general, but whether expert and peer labels are associated with overconfidence in particular types of behavioral errors, namely under-editing and over-editing. For this reason, calibration bias and absolute calibration error were used as the primary measures of metacognitive miscalibration. We nevertheless recognize that relative accuracy and metacognitive sensitivity are important dimensions of language judgment, and future research could examine whether authority cues also affect metacognitive resolution using confidence–accuracy calibration curves, type-2 ROC analyses, or meta-*d*′-based measures.

Authority cues may be one such source of systematic distortion. In persuasion research, self-validation theory argues that individuals do not only generate thoughts; they also evaluate the validity of those thoughts (Petty et al., 2002; Briñol and Petty, 2009). Source credibility can influence this secondary appraisal by increasing or decreasing confidence in one's own cognitive responses (Tormala et al., 2006). Applied to linguistic judgment, this suggests that expertise cues may not simply change whether participants like or dislike a translation. They may change how much participants trust their own language judgments. For example, when a translation is labeled as produced by an expert, participants may become less confident in challenging the original wording, even when they detect a potential error. In contrast, when the same translation is labeled as produced by a student, participants may become more confident in their own corrections, even when the original wording is acceptable.

This mechanism is best described as authority-induced metacognitive miscalibration. It differs from ordinary source credibility effects because the key outcome is not only perceived credibility of the source, but also the alignment between the participant's confidence and actual judgment accuracy. In an English–Chinese revision paradigm, this can be measured by comparing confidence ratings with objective correctness at the segment or decision level. If participants show high confidence in failing to revise erroneous expert-labeled segments, this would indicate confidence inflation in non-revision decisions. If participants show high confidence in revising acceptable student-labeled segments, this would indicate confidence inflation in unnecessary revision decisions. Both patterns reflect metacognitive miscalibration because confidence no longer tracks accuracy.

The following hypothesis therefore specifies the proposed psychological mechanism:

**H3**. Authority cues will induce metacognitive miscalibration in linguistic judgment. Expert cues will increase confidence in non-revision decisions, including cases where the original translation contains objective errors, whereas peer cues will increase confidence in revision decisions, including cases where the original translation is objectively acceptable.

### 2.4 Over-editing and under-editing as behavioral manifestations of miscalibration

If authority cues distort metacognitive calibration, they should also affect behavior. Translation revision is well suited to testing this prediction because revision decisions can be behaviorally observed. A participant may leave a segment unchanged, make a necessary correction, make an unnecessary correction, or fail to correct an existing error. These outcomes allow the study to distinguish between two forms of maladaptive behavioral control: over-editing and under-editing.

Over-editing refers to revising a segment that is already acceptable. It is not simply a high number of edits; it is an unnecessary intervention that does not improve the target text and may even reduce its quality. Under-editing refers to failing to revise a segment that contains an objective error. It is not simply low revision effort; it is an omission of a needed correction. The distinction is important because an increase in total revision count does not necessarily indicate better performance. A psychologically meaningful analysis should examine whether revisions are appropriate relative to the objective status of the segment.

Research on revision and post-editing has increasingly emphasized the importance of process and product indicators. The boundary between revision and post-editing has been discussed in relation to industry practice, cognitive effort, and quality control (Koponen et al., 2020). Studies of post-editing performance typically measure both process data, such as time, pauses, and editing operations, and product data, such as accuracy, fluency, and error correction (Peng et al., 2024). Similarly, translation revision studies have used eye tracking, screen recording, and product assessment to connect decision processes with revision outcomes (Cao et al., 2023). These approaches support the present study's use of revision behavior as a behavioral indicator of language judgment.

However, the present study adds a psychological layer to these behavioral measures. It does not ask only whether participants revise more or less under different labels. Instead, it asks whether their revision behavior becomes misaligned with objective need because of authority-induced metacognitive miscalibration. Expert cues may raise the threshold for intervention: participants may assume that a professional translator's rendering is likely correct and therefore refrain from correcting genuine errors. Peer cues may lower the threshold for intervention: participants may assume that a student translator's rendering is likely flawed and therefore modify acceptable segments. These two patterns correspond to under-editing and over-editing, respectively.

This leads to two behavioral hypotheses:

**H4a**. Expert cues will increase under-editing, reflected in a higher likelihood of failing to revise objectively erroneous translation segments.

**H4b**. Peer cues will increase over-editing, reflected in a higher likelihood of revising objectively acceptable translation segments.

### 2.5 Integrating authority cues, metacognitive miscalibration, and revision behavior

The preceding sections suggest an integrated psychological model. Authority cues first alter perceived source credibility. Perceived credibility then affects linguistic judgment by changing how participants evaluate the target text and how much they trust their own decisions. This process produces metacognitive miscalibration when confidence no longer corresponds to actual judgment accuracy. Finally, miscalibration manifests behaviorally as over-editing or under-editing.

This model contributes to three areas of psychological research. First, it extends source credibility theory from persuasive communication to linguistic judgment. Prior research has shown that source credibility can shape message evaluation (Hovland and Weiss, 1951; Pornpitakpan, 2004), but less is known about how expertise cues influence confidence calibration in language-based decision tasks. Second, it extends metacognitive confidence research by examining whether social authority cues can distort confidence–accuracy alignment in a linguistically complex task. Existing metacognition research has largely focused on perception, memory, and decision tasks (Maniscalco and Lau, 2012; Fleming and Lau, 2014; Fleming, 2024); the present study applies this framework to language judgment. Third, it extends translation-process research by treating revision behavior as a behavioral expression of metacognitive regulation rather than only as a measure of translation competence.

The study also differs from recent source-labeling research in post-editing. (Cao and Zhou 2026) demonstrated that perceived human vs. AI source labels can shape evaluation and post-editing behavior. The present study shifts the theoretical focus from human–AI attribution to human–human authority attribution and from general cognitive bias to metacognitive miscalibration. This distinction is important for positioning the study within psychology. The key research question is not whether professional labels influence translation performance, but whether authority cues disrupt the calibration between confidence and accuracy in linguistic judgment.

The final hypothesis therefore specifies a statistical mediation expectation rather than a definitive claim about temporal causality:

**H5**. Metacognitive miscalibration will statistically account for part of the relationship between authority cues and revision behavior. Specifically, expert cues will be associated with increased under-editing partly through inflated confidence in non-revision decisions, whereas peer cues will be associated with increased over-editing partly through inflated confidence in revision decisions.

**Figure 1:**
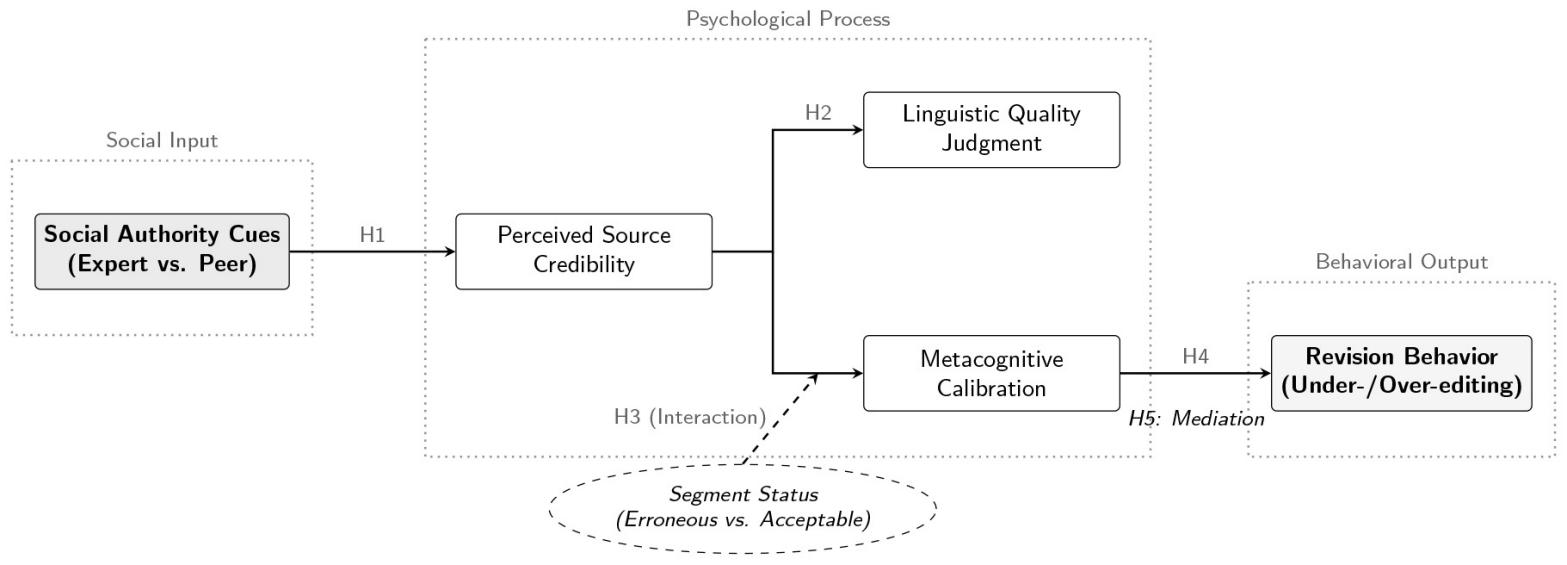
Theoretical framework of authority-induced metacognitive miscalibration. The model illustrates how social authority cues influence behavioral outcomes (under-editing and over-editing) through the mediating roles of perceived source credibility and metacognitive calibration, while accounting for the interactive effect of segment status.

The complete theoretical framework, encompassing all proposed hypotheses (H1 to H5), is illustrated in Figure 1. These hypotheses define the present study as a psychology-of-language investigation. English–Chinese translation revision is used as an experimental paradigm, but the theoretical target is broader: how social authority cues reshape metacognitive calibration and behavioral control during linguistic judgment.

## 3 Materials and methods

### 3.1 Study design

This study employed a controlled behavioral experiment using an English–Chinese revision paradigm. The experiment was designed to test whether source identity labels, used here as authority cues, affect metacognitive calibration in linguistic judgment and whether such effects are expressed behaviorally as over-editing or under-editing. The study used a 3 × 2 within-subject design. The first factor was authority cue, with three levels: expert translator, peer translator, and anonymous source. The second factor was segment status, with two levels: objectively erroneous segment and objectively acceptable segment. Importantly, the authority labels were experimentally assigned and did not reflect the actual origin of the translations; therefore, the manipulation concerned perceived source identity rather than genuine differences in translator expertise.

Authority cues were manipulated at the text level. Each participant reviewed 12 English–Chinese translation texts. Four texts were presented with an expert-translator label, four with a peer-translator label, and four with an anonymous-source label. Each text contained six target segments: three objectively erroneous segments and three objectively acceptable segments. Thus, each participant completed 72 segment-level revision decisions, consisting of 36 erroneous segments and 36 acceptable segments. For each participant, each authority-cue condition contributed 24 segment-level observations, including 12 erroneous and 12 acceptable segments. Because the translation materials were held constant across the experimental lists, any systematic differences across the expert, peer, and anonymous conditions could be interpreted as effects of the assigned source label rather than differences in the objective quality of the translation materials.

The experiment used three counterbalanced stimulus lists. In each list, every participant saw each text only once. Across the three lists, each text appeared once in the expert condition, once in the peer condition, and once in the anonymous condition. Participants were randomly assigned to one of the three lists, with 60 participants assigned to each list. This counterbalancing procedure ensured that differences between authority-cue conditions could not be attributed to the intrinsic difficulty, topic, or quality of any particular text. Segment status was not disclosed to participants, so participants made their revision decisions and confidence judgments without knowing whether a target segment had been pre-classified as objectively erroneous or objectively acceptable.

### 3.2 Participants

A total of 191 Chinese university students entered the experiment. Eleven participants were excluded before analysis according to the pre-specified exclusion criteria: four did not complete the full experiment, three failed the attention-check item, two scored below 50% on the final source-label recognition check, and two reported full-time professional translation experience. The final analytic sample consisted of 180 participants.

All participants were native speakers of Mandarin Chinese and were enrolled in English-major, translation-major, or related language-program courses at Chinese universities. All participants had completed at least one university-level English–Chinese translation course before the experiment. Participants were excluded if they reported full-time professional translation experience, a history of neurological disorder, uncorrected visual impairment, or a diagnosed reading disorder.

To address potential heterogeneity in language-related background, we recorded participants' self-rated English proficiency, number of completed translation courses, and prior revision-task experience. These variables were not used to define separate experimental groups because the primary aim of the study was to test within-subject effects of experimentally assigned source labels. However, they were used as covariates in sensitivity analyses to examine whether the main behavioral findings remained stable after accounting for individual differences in English proficiency and translation-related experience.

No participant was informed of the authority-cue hypothesis before the task. Participants were told that the study examined English–Chinese revision decisions and confidence in language judgment. Because the source labels were experimentally assigned, all participants received a written debriefing after the experiment explaining that the labels were used only for experimental manipulation and did not indicate the actual identity of the translator.

### 3.3 Sample size rationale

The target sample size was determined before data collection by the counterbalanced within-subject design and practical feasibility rather than by a formal a priori power analysis. We targeted a final sample of 180 participants so that 60 participants could be assigned to each of the three counterbalanced stimulus lists. This allocation ensured that each of the 12 texts appeared equally often under the expert, peer, and anonymous label conditions across participants. With 180 participants, the design yielded 2,160 text-level observations for perceived source credibility and linguistic quality judgments and 12,960 segment-level observations for revision decisions, confidence ratings, calibration outcomes, over-editing, and under-editing.

We do not treat the 12,960 segment-level observations as statistically independent substitutes for 12,960 participants. Because decisions were repeated within participants and crossed with text and segment items, statistical precision in this design depends on the number of participants, the number of items, the repeated-measures structure, and the relevant random-effects variance components. For this reason, all confirmatory segment-level analyses used mixed-effects models with crossed random intercepts for participant and target segment, and all text-level analyses used crossed random intercepts for participant and text.

No formal a priori simulation-based power analysis was conducted before data collection. Therefore, the sample size should be understood as design- and feasibility-based, with balanced assignment across the three stimulus lists. To address the adequacy of the final design, we conducted a revision-stage simulation-based sensitivity power analysis for the primary mixed-effects models.

### 3.4 Materials and authority-cue manipulation

The experimental materials consisted of 12 English source texts and their corresponding Chinese translations. Each English source text contained 100 words. The texts covered neutral topics, including education, daily communication, environmental awareness, reading habits, technology use, public services, workplace routines, cultural exchange, travel planning, consumer behavior, health information, and urban life. The texts did not contain emotionally arousing, politically sensitive, or specialized technical content. No machine-translation output was used in constructing the materials.

For each English source text, a Chinese target translation was prepared and then edited to contain six target segments. Three target segments were objectively erroneous and required revision. Three target segments were objectively acceptable and required no revision. The 36 erroneous segments were balanced across three error types: 12 semantic mistranslation errors, 12 omission or addition errors, and 12 terminology or collocation errors. The 36 acceptable segments were semantically accurate, contextually appropriate, and fluent in Chinese. Non-target portions of the translations were retained to provide context but were not included in the confirmatory segment-level analyses.

Three bilingual translation experts independently validated the materials before the formal experiment. Each expert had a doctoral degree or equivalent professional experience in translation studies, applied linguistics, or English–Chinese translation. The experts evaluated each target segment for objective status, error type, and expected correction. A segment was retained only when all three experts agreed on whether it was erroneous or acceptable. For erroneous segments, the experts also agreed on the intended error type and the minimum correction required for a participant's revision to be coded as accurate. The final set of 72 target segments achieved unanimous expert agreement.

Authority cues were presented as translator identity labels. The following English versions report the labels used in the experiment; the labels were presented to participants in Chinese:

**Expert condition:** This Chinese translation was produced by a senior professional translator with more than 10 years of English–Chinese translation experience.

**Peer condition:** This Chinese translation was produced by an undergraduate translation student.

**Anonymous condition:** No information about the translator is available.

The labels were assigned experimentally through the counterbalanced stimulus lists. They did not describe the actual origin of the translation. The objective translation material was held constant across lists, and only the authority cue varied across participants.

### 3.5 Experimental procedure

The experiment was implemented as a browser-based behavioral task using jsPsych (de Leeuw, 2015; de Leeuw et al., 2023). All sessions were conducted in university computer laboratories. Participants completed the task individually on desktop computers. Internet access, copy-and-paste functions, electronic dictionaries, translation software, and external reference tools were disabled during the experiment.

Each session followed the same sequence. First, participants read the consent form and completed a background questionnaire. The questionnaire recorded age, gender, native language, English-learning history, number of completed translation courses, self-rated English proficiency, and prior revision-task experience. These variables were used for sample description and sensitivity checks, not for defining the primary hypothesis tests.

Second, participants read the task instructions. The instructions stated that they would review Chinese translations of English texts, decide whether selected Chinese segments should be revised, and report their confidence in each decision. Participants were instructed to revise only when they believed that a revision was necessary to improve accuracy, completeness, terminology, collocation, or fluency. They were also instructed that not every marked segment contained an error.

Third, participants completed two practice texts that were not included in the formal analysis. The practice phase familiarized participants with the display format, the revision interface, the binary revise-or-keep decision, the text-input box for revisions, and the 0–100 confidence scale. Participants proceeded to the formal task only after completing both practice texts.

The formal task consisted of 12 text blocks. At the beginning of each block, participants saw the English source text, the full Chinese translation, and the translator identity label. Immediately after reading the text and label, participants rated perceived source credibility and overall translation quality. They then completed six segment-level revision trials for that text. In each trial, the English source sentence, the relevant Chinese target segment, and its surrounding Chinese context were displayed. Participants selected either “keep unchanged” or “revise.” If they selected “revise,” they typed a revised Chinese version in a text box. After each segment-level decision, participants rated their confidence in the correctness of that decision on a 0–100 scale.

The order of the 12 texts was randomized for each participant, with the restriction that no more than two consecutive texts appeared under the same authority-cue condition. The six target segments within each text were presented in their original textual order to preserve discourse coherence. At the end of the experiment, participants completed a source-label recognition check for the 12 texts and one attention-check item. Participants then received a written debriefing explaining the purpose of the authority-cue manipulation.

### 3.6 Measures

#### 3.6.1 Authority cue

Authority cue was the experimentally manipulated independent variable. It had three levels: expert, peer, and anonymous. In statistical models, anonymous served as the reference condition unless otherwise specified.

#### 3.6.2 Segment status

Segment status was an item-level property determined during expert validation. Each target segment was coded as either objectively erroneous or objectively acceptable. Erroneous segments required revision. Acceptable segments required no revision.

#### 3.6.3 Perceived source credibility

Perceived source credibility was measured at the text level using three items: “The translator is competent,” “The translation source is credible,” and “The translator is professionally reliable.” Participants rated each item on a 7-point Likert scale ranging from 1 = strongly disagree to 7 = strongly agree. The three items were averaged to form a perceived source credibility score. Internal consistency was evaluated using Cronbach's alpha (Cronbach, 1951).

#### 3.6.4 Linguistic quality judgment

Linguistic quality judgment was measured at the text level using three 7-point ratings: accuracy, fluency, and overall quality. Higher scores indicated more favorable judgments. The three items were averaged to form the overall linguistic quality judgment score.

#### 3.6.5 Revision decision

Revision decision was measured at the segment level. A decision was coded as 0 when the participant selected “keep unchanged” and as 1 when the participant selected “revise.” When a participant selected “revise” but entered an empty text box, the response was coded as an attempted revision and was treated as inaccurate.

#### 3.6.6 Decision accuracy

Decision accuracy was coded at the segment level. For objectively erroneous segments, accuracy was coded as 1 only when the participant revised the segment and the revision corrected the target error without introducing a new meaning error. If the participant left an erroneous segment unchanged or revised it without correcting the target error, accuracy was coded as 0. For objectively acceptable segments, accuracy was coded as 1 when the participant kept the segment unchanged and as 0 when the participant revised it. This coding scheme aligned decision accuracy with the objective need for revision.

#### 3.6.7 Confidence

Confidence was measured at the segment level immediately after each revision decision. Participants responded to the question: “How confident are you that your decision for this segment is correct?” The scale ranged from 0 = not confident at all to 100 = completely confident. For analysis, confidence was rescaled to the interval from 0 to 1.

#### 3.6.8 Metacognitive miscalibration

Metacognitive miscalibration was operationalized as the discrepancy between confidence and actual decision accuracy. This operationalization was selected because the primary theoretical question of the present study concerns whether authority cues produce directional overconfidence or underconfidence relative to the objective correctness of each segment-level revision decision. For participant *i* and segment *j*, scaled confidence was denoted as Confidence_*ij*_ and decision accuracy as Accuracy_*ij*_.

Directional calibration bias was calculated as:


$${\text{Calibration Bias}}_{ij}={\text{Confidence}}_{ij}-{\text{Accuracy}}_{ij}.$$

Positive values indicated overconfidence, whereas negative values indicated underconfidence. This measure provides a direct segment-level estimate of whether confidence is higher or lower than warranted by objective decision accuracy. Because each participant made repeated decisions across multiple authority-cue and segment-status conditions, this segment-level measure could be analyzed using mixed-effects models with crossed participant and item random effects.

Absolute calibration error was calculated as:


$${\text{Absolute Calibration Error}}_{ij}=|{\text{Confidence}}_{ij}-{\text{Accuracy}}_{ij}|.$$

Higher absolute calibration error indicated a larger discrepancy between confidence and accuracy regardless of direction. Thus, calibration bias captured the direction of miscalibration, whereas absolute calibration error captured the magnitude of miscalibration.

We recognize that other metacognitive measures are also available. Confidence–accuracy calibration curves can describe how observed accuracy changes across confidence levels, and signal-detection-based measures, such as type-2 ROC or meta-*d*′-based indices, can assess metacognitive sensitivity or relative accuracy, namely the extent to which confidence discriminates correct from incorrect decisions. These approaches are valuable, but they address a somewhat different question from the primary focus of the present study. Our hypotheses focused on whether expert and peer labels were associated with inflated confidence in specific types of incorrect revision decisions, such as under-editing erroneous segments or over-editing acceptable segments. For this reason, directional calibration bias and absolute calibration error were selected as the primary measures of metacognitive miscalibration in the confirmatory analyses.

#### 3.6.9 Over-editing

Over-editing was coded only for objectively acceptable segments. It was coded as 1 when an acceptable segment was revised and the revision was not required for semantic accuracy, completeness, terminology, collocation, or fluency. It was coded as 0 when the acceptable segment was kept unchanged. In the primary analysis, any unnecessary revision of an expert-validated acceptable segment was treated as over-editing.

#### 3.6.10 Under-editing

Under-editing was coded only for objectively erroneous segments. It was coded as 1 when an erroneous segment was left unchanged or revised without correcting the target error. It was coded as 0 when the participant's revision corrected the target error without introducing a new meaning error.

### 3.7 Coding and data quality control

Participant-entered revisions were coded by two independent bilingual coders. Both coders were trained using a coding manual before coding the formal responses. The manual specified the objective status of each target segment, the intended error type for erroneous segments, the minimum correction required for an accurate revision, and the criteria for coding over-editing and under-editing. Coders were blind to the authority-cue condition and participant identity.

For erroneous segments, coders evaluated whether the submitted revision corrected the target error. A revision was coded as accurate only when it corrected the intended error and did not introduce a new semantic error. For acceptable segments, coders evaluated whether the submitted revision was necessary. A revision was coded as over-editing when it altered an already acceptable segment without improving accuracy, completeness, terminology, collocation, or fluency. Inter-coder reliability was assessed using Cohen's kappa for binary coding decisions, following standard practice for observational coding reliability (Hallgren, 2012). Coding disagreements were resolved by a third bilingual adjudicator. The adjudicated codes were used in all analyses.

The pre-specified participant-level exclusion criteria were applied before hypothesis testing. Participants were excluded if they did not complete the full experiment, failed the attention-check item, scored below 50% on the final source-label recognition check, completed the task in less than one-third of the sample median completion time, reported full-time professional translation experience, or had missing values on the core task variables. The final analytic sample included only participants who satisfied all inclusion criteria and passed all data-quality checks. Because the experimental interface required a response for each segment-level decision and confidence rating, the task dataset contained complete segment-level observations for all included participants.

### 3.8 Statistical analysis

All analyses were conducted in R 4.6.0 (R Core Team, 2026). Linear mixed-effects models and generalized linear mixed-effects models were fitted using the lme4 package (Bates et al., 2015). Tests for linear mixed-effects models were obtained using the lmerTest package (Kuznetsova et al., 2017). Estimated marginal means and pairwise contrasts were computed using estimated marginal means procedures (Lenth, 2016). Model-based mediation analyses were conducted using mediation procedures (Imai et al., 2010; Tingley et al., 2014). Because confidence ratings and revision decisions were collected within the same segment-level task and in close temporal proximity, these mediation analyses were interpreted as tests of indirect statistical association rather than as definitive evidence of a temporally ordered causal mechanism. The analytic strategy followed standard recommendations for repeated-measures linguistic and psycholinguistic data with crossed participant and item structures (Baayen et al., 2008). Logistic mixed-effects models were used for binary outcomes rather than applying linear models to proportions (Jaeger, 2008).

All hypothesis tests were two-tailed with α = 0.05. For families of pairwise contrasts within the same outcome, *p* values were adjusted using the Benjamini–Hochberg false discovery rate procedure (Benjamini and Hochberg, 1995). Continuous outcomes were analyzed with linear mixed-effects models. Binary outcomes were analyzed with logistic mixed-effects models using a logit link. Participant and target segment were treated as crossed random intercepts in segment-level analyses. Participant and text were treated as crossed random intercepts in text-level analyses.

For H1, perceived source credibility was analyzed at the text level using the following linear mixed-effects model:


$${\text{Credibility}}_{it}=\beta_{0}+\beta_{1}{\text{Expert}}_{it}+\beta_{2}{\text{Peer}}_{it}+u_{i}+v_{t}+\epsilon_{it},$$

where *i* indexed participant and *t* indexed text. The anonymous condition served as the reference category. The primary tests were the expert-vs.-anonymous contrast and the peer-vs.-anonymous contrast.

For H2, linguistic quality judgment was analyzed using the same text-level model structure:


$${\text{Quality}}_{it}=\beta_{0}+\beta_{1}{\text{Expert}}_{it}+\beta_{2}{\text{Peer}}_{it}+u_{i}+v_{t}+\epsilon_{it}.$$

This model tested whether authority cues biased quality judgments independently of the objective text material.

For H3, metacognitive miscalibration was analyzed at the segment level. Two outcomes were modeled separately: directional calibration bias and absolute calibration error. The confirmatory model included authority cue, segment status, and their interaction:


$$\begin{array}{cc} {\text{Miscalibration}}_{ij}= & \beta_{0}+\beta_{1}{\text{Expert}}_{ij}+\beta_{2}{\text{Peer}}_{ij}+\beta_{3}{\text{Erroneous}}_{ij}\\ +\beta_{4}\left({\text{Expert}}_{ij}\times {\text{Erroneous}}_{ij}\right)\\ +\beta_{5}\left({\text{Peer}}_{ij}\times {\text{Erroneous}}_{ij}\right)+u_{i}+w_{j}+\epsilon_{ij}. \end{array}$$

where *j* indexed target segment. The acceptable-segment and anonymous-source condition served as the reference category. The interaction terms tested whether the effect of authority cue differed between objectively erroneous and objectively acceptable segments.

For H4a, under-editing was analyzed only among objectively erroneous segments. The binary outcome was coded as 1 for under-editing and 0 for accurate correction. The logistic mixed-effects model was:


$$\text{logit}\left\{P\left({\text{UnderEditing}}_{ij}=1\right)\right\}=\beta_{0}+\beta_{1}{\text{Expert}}_{ij}+\beta_{2}{\text{Peer}}_{ij}+u_{i}+w_{j}.$$

The key test for H4a was whether the expert condition increased the odds of under-editing relative to the anonymous condition.

For H4b, over-editing was analyzed only among objectively acceptable segments. The binary outcome was coded as 1 for over-editing and 0 for keeping the segment unchanged. The logistic mixed-effects model was:


$$\text{logit}\left\{P\left({\text{OverEditing}}_{ij}=1\right)\right\}=\beta_{0}+\beta_{1}{\text{Expert}}_{ij}+\beta_{2}{\text{Peer}}_{ij}+u_{i}+w_{j}.$$

The key test for H4b was whether the peer condition increased the odds of over-editing relative to the anonymous condition.

For H5, two mediation paths were tested. The first path tested whether metacognitive miscalibration mediated the relationship between expert cues and under-editing in erroneous segments. The second path tested whether metacognitive miscalibration mediated the relationship between peer cues and over-editing in acceptable segments. In both paths, authority cue was the treatment variable, calibration bias was the mediator, and revision behavior was the outcome. Indirect effects were estimated using 5,000 cluster-bootstrap resamples at the participant level. The mediation effect was considered statistically supported when the 95% bootstrap confidence interval for the indirect effect did not include zero.

Model estimates were reported as regression coefficients for linear mixed-effects models and odds ratios for logistic mixed-effects models. All estimates were reported with 95% confidence intervals. Descriptive statistics were reported as means and standard deviations for continuous variables and as frequencies and percentages for categorical variables.

## 4 Results

### 4.1 Participant characteristics

Table 1 presents the participant characteristics and task-level descriptive statistics. The final analytic sample included 180 participants. The mean age was 21.43 years, and 134 participants were female. Participants had learned English for an average of 13.1 years and had completed an average of 3.2 translation courses. Their mean self-rated English proficiency was 4.58 on a 7-point scale. Each participant completed 72 segment-level revision decisions, producing 12,960 observations in total. The dataset contained an equal number of erroneous-segment and acceptable-segment observations. The overall revision rate was 48.2%, and the overall decision accuracy was 68.8%. Mean confidence was 0.753 after rescaling to the 0–1 range, indicating that participants were generally confident in their revision decisions. The positive mean directional calibration bias suggested mild overall overconfidence. Inter-coder reliability was high for both revision accuracy coding and over-/under-editing coding, with Cohen's κ values of 0.88 and 0.83, respectively.

**Table 1:** Participant characteristics and task-level descriptive statistics.

Variable	Mean/*n*	SD/%
Participant characteristics		
Final analytic sample	180	–
Age	21.43	1.85
Female participants	134	74.4%
Years of English learning	13.1	2.26
Completed translation courses	3.2	1.38
Self-rated English proficiency^a^	4.58	0.91
15.6-2.4,-1.3243ptPrior revision-task experience	46	25.6%
Task-level descriptive statistics		
Segment-level observations	12,960	–
Erroneous-segment observations	6,480	50.0%
Acceptable-segment observations	6,480	50.0%
Overall revision rate	6,247	48.2%
Overall decision accuracy	8,916	68.8%
Mean confidence score^b^	0.753	0.142
Directional calibration bias^b^	0.065	0.211
Absolute calibration error^b^	0.287	0.163
Cohen's κ for revision accuracy coding	0.88	–
Cohen's κ for over-/under-editing coding	0.83	–

a Self-rated English proficiency was measured on a 7-point Likert scale (1 = very poor, 7 = excellent).

b Descriptive statistics for mean confidence score, directional calibration bias, and absolute calibration error were calculated based on participant-level aggregated scores (*N* = 180) to accurately reflect between-subject variability. Confidence scores were rescaled from 0–100 to 0–1. Directional calibration bias was calculated as confidence minus decision accuracy. Absolute calibration error was calculated as the absolute difference between confidence and decision accuracy.

### 4.2 Manipulation check: perceived source credibility

**Figure 2:**
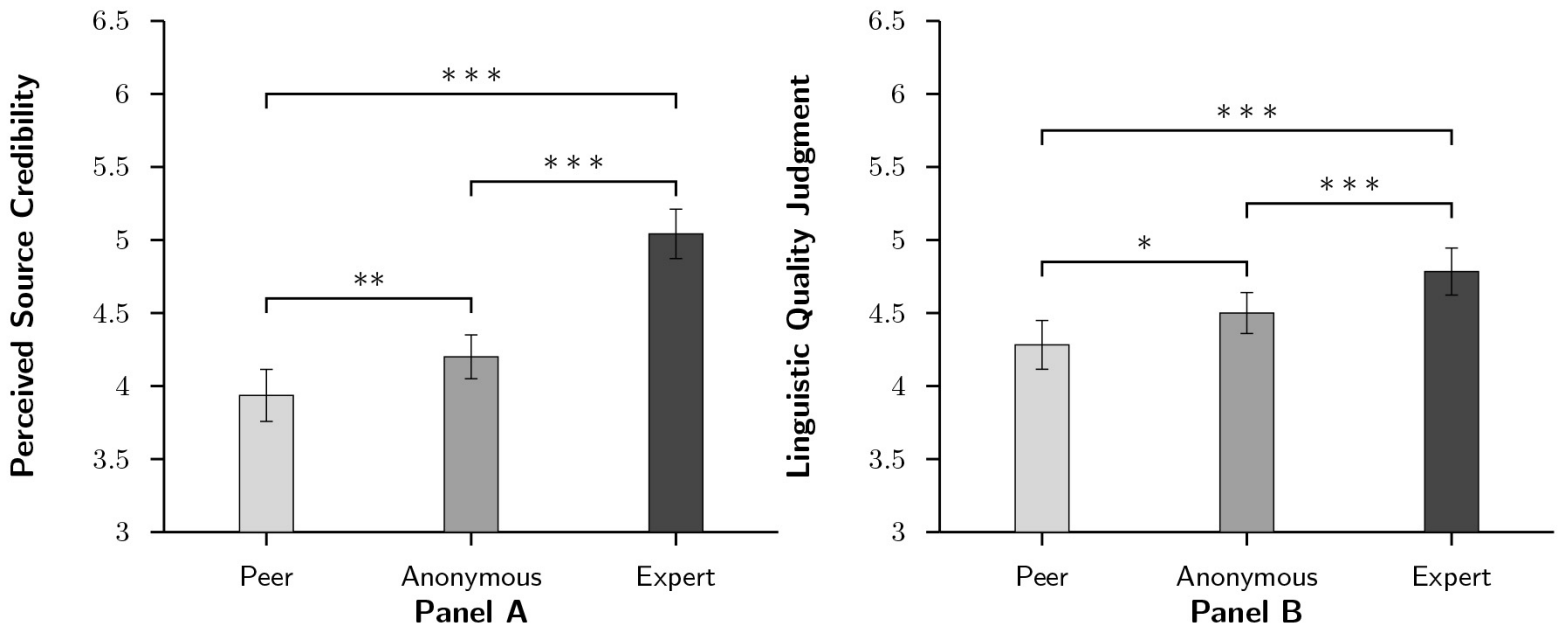
Model-estimated authority-cue effects on perceived source credibility and linguistic quality judgment. Error bars represent 95% confidence intervals. Significance levels: ^*^p < 0.05, ^**^p < 0.01, ^***^p < 0.001.

The authority-cue manipulation successfully changed participants' perceived source credibility. As shown in Table 2 and Figure 2, translations labeled as produced by a senior professional translator received significantly higher source credibility ratings than anonymous translations, *b* = 0.842, 95% CI [0.673, 1.011], *p* < 0.001. In contrast, translations labeled as produced by a peer translator received lower source credibility ratings than anonymous translations, *b* = −0.264, 95% CI [−0.442, −0.086], *p* = 0.004. The expert condition also differed significantly from the peer condition, *b* = 1.106, 95% CI [0.932, 1.280], *p* < 0.001. These results indicate that the professional translator label functioned as a strong authority cue, whereas the peer translator label reduced perceived source credibility. Thus, the manipulation check supported H1 and confirmed that the experimental labels produced the intended differences in perceived authority.

**Table 2:** Mixed-effects estimates for perceived source credibility and linguistic quality judgment.

Outcome	Contrast	Estimate	SE	95% CI	*p*
Source credibility	Expert vs. anonymous	0.842	0.086	[0.673, 1.011]	< 0.001
	Peer vs. anonymous	–0.264	0.091	[−0.442, −0.086]	0.004
	Expert vs. peer	1.106	0.089	[0.932, 1.280]	< 0.001
Quality judgment	Expert vs. anonymous	0.284	0.082	[0.123, 0.445]	0.001
	Peer vs. anonymous	–0.218	0.085	[−0.385, −0.051]	0.010
	Expert vs. peer	0.502	0.083	[0.339, 0.665]	< 0.001

Estimates are from text-level linear mixed-effects models with crossed random intercepts for participant and text. Anonymous was the reference condition in the primary models. Pairwise contrasts were adjusted using the false discovery rate procedure.

### 4.3 Authority-cue effects on linguistic quality judgment

Authority cues also influenced participants' linguistic quality judgments. As shown in Table 2 and Figure 2, expert-labeled translations received significantly higher quality ratings than anonymous translations, *b* = 0.284, 95% CI [0.123, 0.445], *p* = 0.001. Peer-labeled translations received significantly lower quality ratings than anonymous translations, *b* = −0.218, 95% CI [−0.385, −0.051], *p* = 0.010. The expert condition was also rated significantly higher than the peer condition, *b* = 0.502, 95% CI [0.339, 0.665], *p* < 0.001. Because the translation materials were counterbalanced across cue conditions, these differences indicate that source identity labels biased participants' quality judgments independently of the objective text materials. These findings support H2.

### 4.4 Authority-cue effects on metacognitive miscalibration

**Figure 3:**
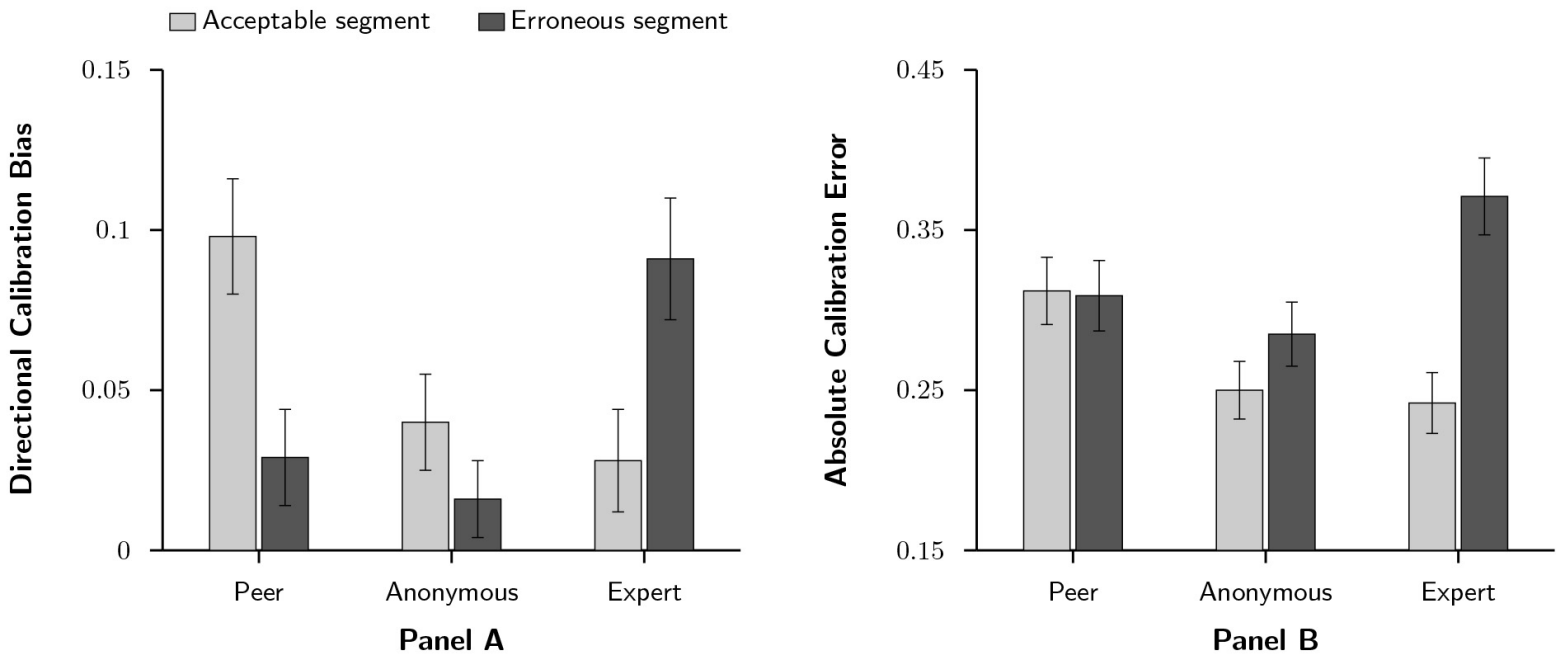
Metacognitive miscalibration by authority cue and segment status. Panel **(A)** shows directional calibration bias (positive values indicate overconfidence). Panel **(B)** shows absolute calibration error. Light bars represent objectively acceptable segments; dark bars represent objectively erroneous segments. Error bars represent 95% confidence intervals.

Table 3 and Figure 3 show the effects of authority cues on metacognitive miscalibration. For directional calibration bias, the peer cue produced a significant positive effect in acceptable segments, *b* = 0.058, 95% CI [0.029, 0.087], *p* < 0.001, indicating greater overconfidence when participants evaluated segments that did not objectively require revision. The expert cue did not significantly affect calibration bias in acceptable segments, *b* = −0.012, 95% CI [−0.043, 0.019], *p* = 0.452. The interaction between expert cue and erroneous segment was significant, *b* = 0.087, 95% CI [0.044, 0.130], *p* < 0.001. This indicates that expert labels increased calibration bias mainly when participants evaluated objectively erroneous segments. The peer cue × erroneous segment interaction was negative, *b* = −0.045, 95% CI [−0.086, −0.004], *p* = 0.031, suggesting that the peer-cue effect on calibration bias was concentrated in acceptable segments rather than erroneous segments. The analysis of absolute calibration error showed the same pattern. Peer-labeled acceptable segments showed greater confidence–accuracy discrepancy than anonymous acceptable segments, *b* = 0.062, 95% CI [0.037, 0.087], *p* < 0.001. The expert cue × erroneous segment interaction was also significant, *b* = 0.094, 95% CI [0.055, 0.133], *p* < 0.001, indicating that expert-labeled erroneous segments produced the largest increase in absolute miscalibration. These findings support H3 and show that authority cues distorted metacognitive calibration in different directions depending on the objective status of the segment.

**Table 3:** Mixed-effects models for metacognitive miscalibration.

Outcome	Predictor	Estimate	SE	95% CI	*p*
Calibration bias	Expert	–0.012	0.016	[−0.043, 0.019]	0.452
	Peer	0.058	0.015	[0.029, 0.087]	< 0.001
	Erroneous segment	–0.024	0.018	[−0.059, 0.011]	0.182
	Expert × erroneous segment	0.087	0.022	[0.044, 0.130]	< 0.001
	Peer × erroneous segment	–0.045	0.021	[−0.086, −0.004]	0.031
Absolute calibration error	Expert	–0.008	0.014	[−0.035, 0.019]	0.562
	Peer	0.062	0.013	[0.037, 0.087]	< 0.001
	Erroneous segment	0.035	0.017	[0.002, 0.068]	0.038
	Expert × erroneous segment	0.094	0.020	[0.055, 0.133]	< 0.001
	Peer × erroneous segment	–0.038	0.019	[−0.075, −0.001]	0.044

Estimates are from segment-level linear mixed-effects models with crossed random intercepts for participant and target segment. Anonymous and acceptable segment served as the reference categories. Calibration bias was calculated as confidence minus decision accuracy. Absolute calibration error was calculated as the absolute value of the confidence–accuracy difference.

### 4.5 Over-editing and under-editing

**Figure 4:**
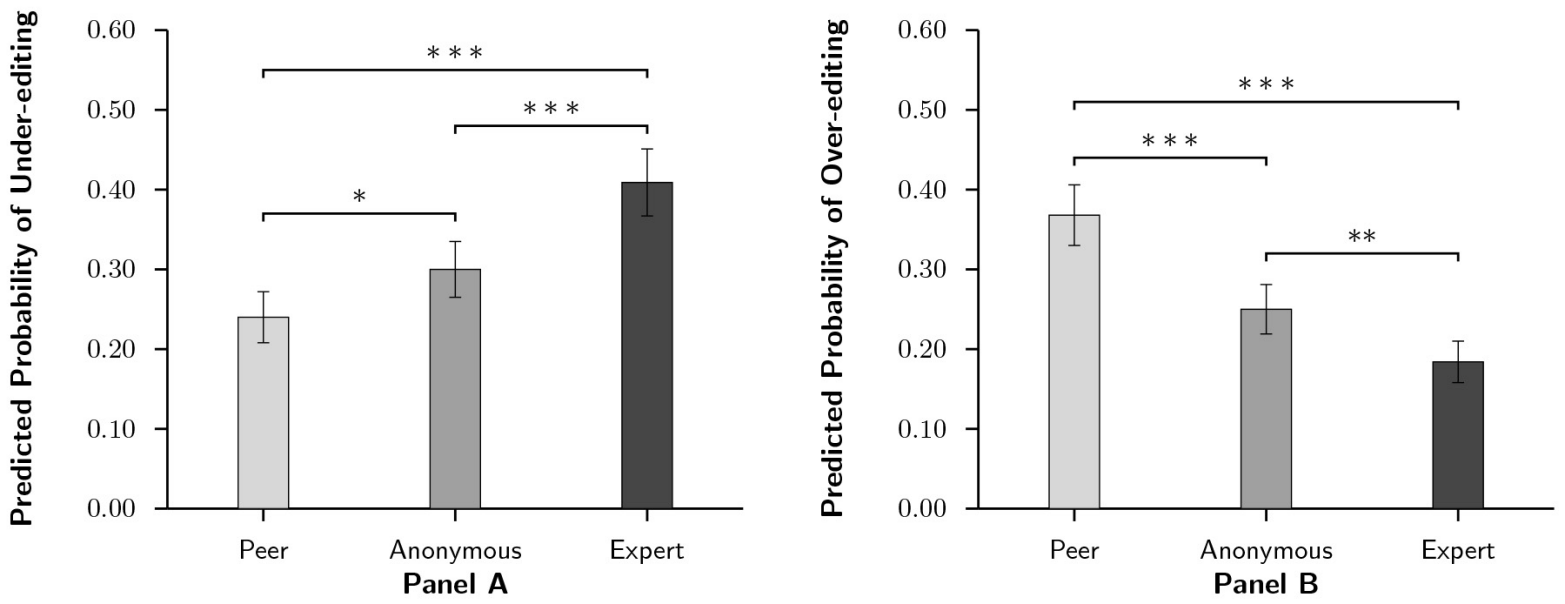
Predicted probabilities of under-editing in erroneous segments [Panel **(A)**] and over-editing in acceptable segments [Panel **(B)**] by authority cue. Error bars represent 95% confidence intervals. Significance levels: ^*^p < 0.05, ^**^p < 0.01, ^***^p < 0.001.

The next analysis examined whether metacognitive miscalibration was reflected in revision behavior. Under-editing was analyzed in objectively erroneous segments, whereas over-editing was analyzed in objectively acceptable segments. The results are reported in Table 4 and Figure 4.

**Table 4:** Logistic mixed-effects models for under-editing and over-editing.

Outcome	Contrast	OR	95% CI	*p*
Under-editing	Expert vs. anonymous	1.62	[1.24, 2.12]	< 0.001
	Peer vs. anonymous	0.74	[0.58, 0.95]	0.018
	Expert vs. peer	2.19	[1.66, 2.89]	< 0.001
Over-editing	Expert vs. anonymous	0.68	[0.51, 0.91]	0.009
	Peer vs. anonymous	1.75	[1.31, 2.34]	< 0.001
	Peer vs. expert	2.57	[1.89, 3.50]	< 0.001

Under-editing was analyzed only in objectively erroneous segments. Over-editing was analyzed only in objectively acceptable segments. Estimates are from logistic mixed-effects models with crossed random intercepts for participant and target segment. OR, odds ratio. Pairwise contrasts were adjusted using the false discovery rate procedure.

#### 4.5.1 Under-editing in erroneous segments

Expert cues significantly increased the likelihood of under-editing in erroneous segments. Compared with the anonymous condition, the expert condition showed higher odds of under-editing, OR = 1.62, 95% CI [1.24, 2.12], *p* < 0.001. In contrast, the peer condition showed lower odds of under-editing than the anonymous condition, OR = 0.74, 95% CI [0.58, 0.95], *p* = 0.018. The expert condition also differed significantly from the peer condition, OR = 2.19, 95% CI [1.66, 2.89], *p* < 0.001. These findings support H4a, indicating that expert authority cues increased the probability that participants failed to correct objectively erroneous segments.

#### 4.5.2 Over-editing in acceptable segments

Peer cues significantly increased the likelihood of over-editing in acceptable segments. Compared with the anonymous condition, the peer condition showed higher odds of over-editing, OR = 1.75, 95% CI [1.31, 2.34], *p* < 0.001. The expert condition showed lower odds of over-editing than the anonymous condition, OR = 0.68, 95% CI [0.51, 0.91], *p* = 0.009. The peer condition also differed significantly from the expert condition, OR = 2.57, 95% CI [1.89, 3.50], *p* < 0.001. These findings support H4b, indicating that peer cues increased unnecessary revision of objectively acceptable segments. These results show two opposite behavioral consequences of authority cues: expert cues promoted excessive restraint in erroneous segments, whereas peer cues promoted excessive intervention in acceptable segments.

### 4.6 Mediation by metacognitive miscalibration

**Figure 5:**
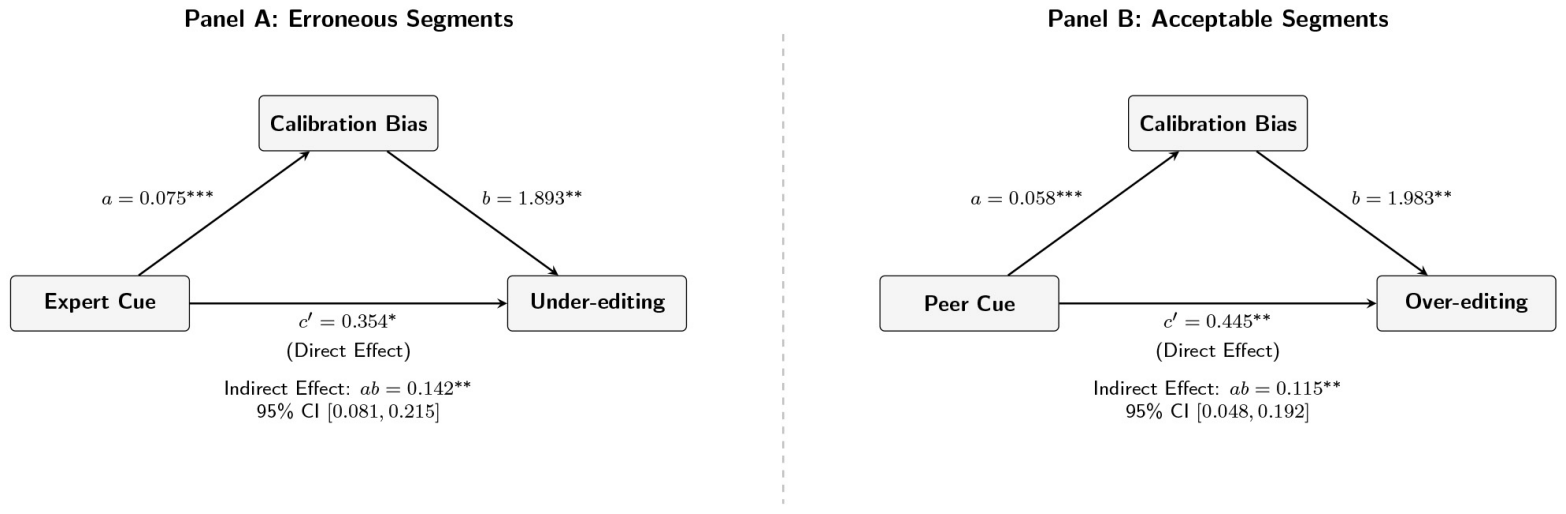
Mediation models of authority-associated metacognitive miscalibration. Panel **(A)** shows the pathway from expert cues to under-editing in objectively erroneous segments. Panel **(B)** shows the pathway from peer cues to over-editing in objectively acceptable segments. Path *a* is presented as an unstandardized linear coefficient. Paths *b* and *c*′ and the indirect effects (*ab*) are reported on the log-odds scale. Significance levels: ^*^p < 0.05, ^**^p < 0.01, ^***^p < 0.001.

The mediation analysis examined whether calibration bias statistically accounted for part of the association between authority cues and revision behavior. As shown in Table 5 and Figure 5, both theoretically specified indirect pathways were statistically supported. In objectively erroneous segments, expert cues were associated with greater calibration bias, and greater calibration bias was in turn associated with a higher likelihood of under-editing. The indirect effect of expert cue on under-editing through calibration bias was significant, *ab* = 0.142, 95% bootstrap CI [0.081, 0.215], *p* = 0.002. This pattern suggests that calibration bias partly accounted for the association between expert cues and under-editing. In objectively acceptable segments, peer cues were associated with greater calibration bias, and greater calibration bias was in turn associated with a higher likelihood of over-editing. The indirect effect of peer cue on over-editing through calibration bias was also significant, *ab* = 0.115, 95% bootstrap CI [0.048, 0.192], *p* = 0.006. This pattern suggests that calibration bias partly accounted for the association between peer cues and over-editing. These findings are consistent with H5. However, because confidence and revision behavior were measured within the same segment-level task and close in time, the mediation results should be interpreted as evidence of a statistically consistent indirect pathway rather than conclusive evidence that metacognitive miscalibration directly caused revision behavior.

**Table 5:** Multilevel mediation results.

Mediation path	Indirect effect	95% bootstrap CI	*p*	Supported
Expert cue → calibration bias → under-editing	0.142	[0.081, 0.215]	0.002	Yes
Peer cue → calibration bias → over-editing	0.115	[0.048, 0.192]	0.006	Yes

The expert-cue mediation path was estimated in objectively erroneous segments. The peer-cue mediation path was estimated in objectively acceptable segments. Indirect effects are reported on the log-odds scale. Indirect effects and confidence intervals were estimated using 5,000 participant-level cluster-bootstrap resamples.

### 4.7 Sensitivity analyses

Sensitivity analyses were conducted to evaluate whether the main behavioral findings were robust to alternative model specifications and coding decisions. As shown in Table 6, the results remained stable across all robustness checks. First, to address possible heterogeneity in participants' language-related background, we refitted the main behavioral models after adjusting for self-rated English proficiency, completed translation courses, and prior revision-task experience. The expert cue continued to increase under-editing in erroneous segments, OR = 1.59, 95% CI [1.22, 2.08], *p* < 0.001. The peer cue also continued to increase over-editing in acceptable segments, OR = 1.72, 95% CI [1.29, 2.30], *p* < 0.001. These results indicate that the main effects of authority cues were not explained by measured differences in English proficiency, translation training, or prior revision-task experience. Second, when a stricter definition of over-editing was applied, the peer-cue effect remained significant, OR = 1.58, 95% CI [1.16, 2.15], *p* = 0.004. Third, after excluding trials in the bottom 10% of the confidence-score distribution, the main effects were unchanged. The expert cue continued to increase under-editing, OR = 1.65, 95% CI [1.25, 2.18], *p* < 0.001, and the peer cue continued to increase over-editing, OR = 1.78, 95% CI [1.32, 2.40], *p* < 0.001. These checks indicate that the main behavioral results were not driven by participant background variables, the operational definition of over-editing, or very low-confidence trials.

**Table 6:** Robustness checks for the effects of authority cues on revision behavior.

	Under-editing (expert vs. anonymous)		Over-editing (peer vs. anonymous)	
Model specification	OR [95% CI]	*p*	OR [95% CI]	*p*
1. Baseline model (Table 4)	1.62 [1.24, 2.12]	< 0.001	1.75 [1.31, 2.34]	< 0.001
2. Covariate-adjusted model^a^	1.59 [1.22, 2.08]	< 0.001	1.72 [1.29, 2.30]	< 0.001
3. Stricter behavioral coding^b^	–	–	1.58 [1.16, 2.15]	0.004
4. Low-confidence exclusion^c^	1.65 [1.25, 2.18]	< 0.001	1.78 [1.32, 2.40]	< 0.001

OR, odds ratio. Under-editing was analyzed in erroneous segments; over-editing was analyzed in acceptable segments.

a Adjusted for self-rated English proficiency, completed translation courses, and prior revision experience.

b Stricter criteria were applied only to the definition of over-editing; under-editing was not re-estimated because its coding definition was unchanged.

c Excluded trials where the confidence score was in the bottom 10% of the distribution.

In addition to the robustness checks reported above, we conducted a revision-stage simulation-based sensitivity power analysis to evaluate whether the final design had adequate sensitivity for detecting the primary authority-cue effects in the mixed-effects models. The analysis was conducted using the simr package (Green and MacLeod, 2016). The simulations preserved the final design structure and random-effects specification, including 180 participants, 12 texts, 72 segment-level decisions per participant, three counterbalanced stimulus lists, and crossed random intercepts for participants and items. Because this analysis was conducted after data collection, it is interpreted as a sensitivity assessment of the final design rather than as a formal a priori power analysis.

As shown in Table 7, the final design achieved estimated power above 80% for all primary target effects examined. For the binary behavioral models, the estimated power was 81.4% for detecting an odds ratio of 1.30 and 97.3% for detecting an odds ratio of 1.50 for the expert effect on under-editing. The estimated power was 83.9% for detecting an odds ratio of 1.30 and 98.1% for detecting an odds ratio of 1.50 for the peer effect on over-editing. For the calibration-bias model, the estimated power was 88.6% for detecting an expert cue × erroneous segment interaction of *b* = 0.05 and 92.7% for detecting a peer effect in acceptable segments of *b* = 0.05. These results suggest that the final design had adequate sensitivity for the primary small-to-moderate authority-cue effects examined in the manuscript.

**Table 7:** Simulation-based sensitivity power analysis for primary mixed-effects models.

Outcome	Target effect	Assumed effect size	Estimated power
Under-editing	Expert vs. anonymous	OR = 1.30	81.4%
Under-editing	Expert vs. anonymous	OR = 1.50	97.3%
Over-editing	Peer vs. anonymous	OR = 1.30	83.9%
Over-editing	Peer vs. anonymous	OR = 1.50	98.1%
Calibration bias	Expert × erroneous segment	*b* = 0.05	88.6%
Calibration bias	Peer effect in acceptable segments	*b* = 0.05	92.7%

Power was estimated using simulation-based procedures for mixed-effects models with 1,000 simulations per condition. The simulations preserved the final design structure and random-effects specification. Because this analysis was conducted after data collection, it is interpreted as a sensitivity assessment of the final design rather than as a formal a priori power analysis.

## 5 Discussion

### 5.1 Authority cues as social inputs to linguistic judgment

This study examined how authority cues shape linguistic judgment through an English–Chinese revision paradigm. The results showed that expert labels increased perceived source credibility, whereas peer labels reduced it. The same authority cues also shifted participants' linguistic quality judgments, even though the translation materials were counterbalanced across conditions. These findings indicate that linguistic judgment is not a purely text-internal evaluation process. Instead, judgments about language can be shaped by socially meaningful cues attached to the linguistic material. Importantly, we do not interpret this pattern as evidence that participants acted irrationally. Expert and peer labels may function as plausible source-based priors under uncertainty, because professional translators are generally expected to make fewer errors than student translators. The key finding is that, when the objective materials were held constant across label conditions, these source-based expectations were associated with systematic shifts in credibility judgments, quality judgments, confidence calibration, and revision behavior.

This interpretation is consistent with classic source credibility research, which shows that people evaluate the same message differently depending on the perceived credibility of the source (Hovland and Weiss, 1951; Pornpitakpan, 2004), and with heuristic-processing accounts suggesting that source cues may guide judgment under uncertainty (Chaiken, 1980; Meinert and Krämer, 2022). Prior research on epistemic vigilance also suggests that people monitor both message content and source reliability when evaluating communicated information (Sperber et al., 2010). Our findings show that this source-monitoring process also operates in a controlled revision task: an expert label encouraged trust in the target text, whereas a peer label encouraged suspicion. Thus, the present study is best interpreted as evidence of heuristic source-based judgment and prior adjustment under uncertainty, rather than as a simple demonstration of irrational authority bias. This result also complements recent post-editing evidence showing that source beliefs can affect editing decisions (Cao and Zhou, 2026). However, the present study shifts the focus from human–machine attribution to human–human authority attribution and from general source-labeling effects to confidence–accuracy calibration in linguistic judgment.

### 5.2 Metacognitive miscalibration as an explanatory pathway

The central contribution of this study is the identification of authority-associated metacognitive miscalibration in linguistic judgment. The results showed that expert cues increased calibration bias and absolute calibration error mainly in erroneous segments, whereas peer cues increased miscalibration mainly in acceptable segments. This pattern suggests that authority cues did not simply make participants more or less confident overall. Rather, they were associated with changes in the alignment between confidence and actual decision accuracy. This finding is consistent with metacognitive theories that treat confidence as a judgment about the reliability of one's own decision (Koriat, 2012; Maniscalco and Lau, 2012; Fleming and Lau, 2014). It also supports recent views that confidence is vulnerable to systematic distortion when decision makers rely on non-diagnostic cues (Fleming, 2024; Rapp and Withall, 2024).

The behavioral results are consistent with this explanatory pathway. Expert cues increased under-editing in erroneous segments, while peer cues increased over-editing in acceptable segments. Thus, the problem was not simply that participants revised more or less; rather, their revision behavior became less aligned with the objective need for revision. This pattern is consistent with self-validation theory, which argues that source-related cues can affect the confidence people place in their own thoughts and judgments (Petty et al., 2002; Tormala et al., 2006). In the present task, expert labels appeared to validate restraint, whereas peer labels appeared to validate intervention. We interpret over-editing and under-editing as behavioral manifestations associated with disrupted metacognitive calibration, rather than as proof that confidence discrepancy directly caused each revision decision.

### 5.3 Implications, limitations, and future directions

These findings have implications for psychology-of-language research and for language-related training. Theoretically, the study shows that linguistic judgment can be influenced by the interaction between social source cues and metacognitive confidence. This extends metacognition research beyond perception, memory, and general decision-making tasks into a linguistically complex judgment setting. Practically, the findings suggest that language and translation training should not focus only on improving error detection or revision accuracy. It should also help learners calibrate their confidence under socially biased conditions. This is consistent with process-oriented translation research showing that revision is a decision-making activity rather than a purely mechanical correction process (Shih, 2015; Cao et al., 2023), and with recent work indicating that learner beliefs and self-efficacy influence revision and post-editing performance (Peng et al., 2024). Training activities that require learners to justify both revisions and non-revisions may help improve confidence calibration, especially when source identity information is present.

Several limitations should be noted. First, although the authority labels were experimentally manipulated, the mediation analysis does not provide the same level of causal evidence as the primary label manipulation. Confidence and revision behavior were measured within the same segment-level task and in close temporal proximity. Therefore, the indirect effects should be interpreted as statistically consistent mediation pathways rather than definitive evidence that metacognitive miscalibration directly caused revision behavior. A stronger design would temporally separate confidence judgment from revision action. For example, future studies could first ask participants to judge whether a segment is correct and report their confidence without revising it immediately, and then ask them in a later phase to decide whether and how to revise the same segment. Such a design would provide a stronger test of the temporal ordering among authority cues, confidence calibration, and revision behavior.

Second, the present study focused on directional calibration bias and absolute calibration error as the primary measures of metacognitive miscalibration. This choice was appropriate for testing whether authority cues were associated with overconfidence or underconfidence in specific segment-level revision decisions. However, these measures do not fully capture all aspects of metacognitive performance. In particular, the present study did not provide a detailed analysis of metacognitive sensitivity or relative accuracy, such as whether confidence ratings discriminated correct from incorrect decisions equally well across authority-cue conditions. Future studies could extend the present findings by using confidence–accuracy calibration curves, type-2 ROC analyses, or meta-*d*′-based measures to examine whether authority cues influence not only calibration bias but also metacognitive resolution.

Third, the target sample size was determined by the counterbalanced experimental design and practical feasibility rather than by a formal a priori power analysis. However, to address the adequacy of the final design, we conducted a revision-stage simulation-based sensitivity power analysis for the primary mixed-effects models. The simulations preserved the final design structure and crossed random-effects specification. The results showed that the final design achieved estimated power above 80% for all primary target effects examined, including the expert effect on under-editing, the peer effect on over-editing, and the authority-cue effects on calibration bias. Although this simulation analysis does not replace a formal a priori power analysis, it suggests that the final design had adequate sensitivity for detecting the small-to-moderate effects targeted in the primary models. Future studies should nevertheless conduct simulation-based power analysis before data collection, with prespecified effect sizes, numbers of participants, numbers of items, and expected variance components.

Fourth, the sample consisted of Chinese university students with English–Chinese translation training, so the findings should not be generalized directly to professional translators or to other language pairs. Fifth, the task used short, controlled texts to ensure experimental validity; future studies should examine whether similar effects occur in longer and more naturalistic revision environments. Sixth, the present study relied on behavioral decisions and confidence ratings. Future work could combine revision tasks with eye tracking, keystroke logging, or screen recording to examine how authority cues alter attention, hesitation, and revision trajectories. Recent evidence suggests that reassessing confidence can improve metacognitive performance (Elosegi et al., 2024), so future studies could also test interventions that prompt revisers to reconsider their confidence before finalizing a revision decision. Such work would clarify whether authority-associated metacognitive miscalibration can be reduced through targeted confidence-calibration training.

## 6 Conclusion

This study examined how authority cues influence linguistic judgment through an English–Chinese revision paradigm. The findings show that participants did not evaluate translations solely on the basis of textual quality. Instead, source labels shaped how credible the translation appeared, how participants judged its quality, and how confident they felt about their own revision decisions. Expert labels encouraged trust and restraint, whereas peer labels encouraged suspicion and intervention. In this sense, authority cues functioned as social inputs to language judgment. The study shows that these cues can disrupt the alignment between confidence and accuracy. When a translation was labeled as expert-produced, participants were more likely to overlook genuine errors; when it was labeled as peer-produced, they were more likely to revise acceptable segments unnecessarily. These findings suggest that over-editing and under-editing are not merely revision habits, but behavioral expressions of metacognitive miscalibration. By treating translation revision as a linguistic judgment task, this study highlights how social authority can shape not only what people decide about language, but also how much they trust their own decisions.

## Data Availability

The original contributions presented in the study are included in the article/supplementary material, further inquiries can be directed to the corresponding authors.
